# High-efficiency encapsulation and pH-triggered release of docetaxel from folic acid-functionalized ZIF-90 nanocarriers

**DOI:** 10.1039/d5ra07118f

**Published:** 2025-11-19

**Authors:** Rizgar Noori, Nian N. N. Maarof, Azad H. Alshatteri

**Affiliations:** a Department of Chemistry, College of Science, Charmo University Chamchamal Kurdistan Region 46023 Iraq Iraq.rzgar.nory@chu.edu.iq; b Department of Chemistry, College of Education, University of Sulaimani Sulaimani 46001 Iraq nian.noori@univsul.edu.iq; c Faculty of Health Sciences, Qaiwan International University Sulaymaniyah Kurdistan Region 46001 Iraq; d Department of Chemistry, College of Education, University of Garmian 46021 Kalar City Kurdistan Region Iraq azadalshatteri@garmian.edu.krd

## Abstract

Metal–organic frameworks (MOFs) offer promising features for drug delivery owing to their high porosity, tunable structures, and surface functionality. Among them, zeolitic imidazolate framework-90 (ZIF-90) is notable for its aldehyde groups, which enable facile functionalization and enhanced biocompatibility. In this work, we report folic acid-modified ZIF-90 nanoparticles as pH-responsive carriers for docetaxel (DTX), a widely used chemotherapeutic agent for breast cancer. The nanocarriers displayed a uniform nanoscale morphology and achieved a high encapsulation efficiency of 92.8% with a drug loading capacity of 8.43%. *In vitro* release studies revealed pronounced pH sensitivity, with accelerated drug release under acidic conditions (pH 5.5), mimicking tumor and endosomal environments, while release was sustained at the physiological pH of 7.4. Kinetic modelling indicated that the Hixson–Crowell and Korsmeyer–Peppas models best described the release, suggesting a combination of surface erosion and anomalous diffusion. Compared with the more widely studied ZIF-8 systems, ZIF-90 offered distinct advantages in enabling stable folic-acid conjugation and enhanced acid-labile degradation. The incorporation of folic acid conferred additional specificity towards folate receptor-overexpressing breast cancer cells, underscoring the translational potential of this platform. To our knowledge, this is among the first systematic studies demonstrating efficient docetaxel encapsulation and controlled release from FA-functionalized ZIF-90, highlighting its promise in further biological evaluations and targeted cancer therapy.

## Abstract 2

Schematic illustration of DTX-loaded ZIF-90/FA nanocarrier synthesis and functionalization.

## Graphical abstract

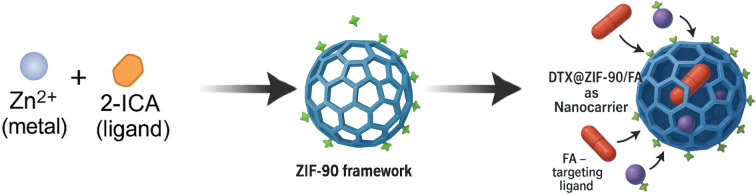

## 1. Introduction

Breast malignancy is the most commonly diagnosed malignancy and the major cause of female cancer fatalities worldwide, accounting for an estimated 2.3 million new cases and 685 000 fatalities in 2022 alone. With increasing world-life-expectancy and lifestyle alterations, the number of cases of breast malignancy will rise exponentially, and accordingly, the incidence will exceed 3 million cases by the year 2040.^1,2^ Despite advancements in early detection, surgical precision, and the advent of molecularly targeted therapeutics, breast cancer mortality remains alarmingly high. This is especially evident in aggressive phenotypes, such as triple-negative breast cancer (TNBC), characterized by the absence of hormone receptors and HER2 overexpression, which severely restricts therapeutic intervention.^3^

Among the chemotherapeutic agents available, docetaxel (DTX) holds prime importance in the treatment of early-stage as well as metastatic breast cancers. Being a semi-synthetic taxane, the drug exploits the stabilizing effect on microtubules, preventing the formation of the mitotic spindle and initiating apoptosis in the fast-dividing tumour cells.^4,5^ Despite its excellent efficacy, its clinical application is hindered by several pharmacokinetic and formulation challenges, including its extremely low aqueous solubility (<0.03 mg mL^−1^), the requirement for toxic excipients (such as polysorbate 80), rapid systemic clearance, and dose-limiting toxicities (including neutropenia, mucositis, and hypersensitivity reactions).^6^ These issues contribute not only to systemic toxicity but also to multidrug resistance (MDR) and suboptimal drug delivery to tumour tissues. Thus, there is an urgent need for advanced drug-delivery systems capable of improving DTX solubility, enhancing the tumour specificity, and enabling sustained and controlled release.

In this context, nanoparticle-based carriers have attracted significant attention for improving the therapeutic index, solubility, pharmacokinetic profile, and tumor-targeting capability of hydrophobic chemotherapeutic agents such as docetaxel. In recent years, multifunctional nanoplatforms that combine drug delivery with tumour-microenvironment modulation to overcome delivery and immunosuppression barriers have emerged.^7^ For instance, a recent study described a ‘dual-warhead’ nanomedicine that concurrently targets extracellular matrix remodeling and immune suppression to sensitize pancreatic ductal adenocarcinoma to immunotherapy.^8^ Such progress illustrates how rationally engineered nanocarriers can be hybridized to possess therapeutic and immunomodulatory capabilities, guiding the rationalization of future hybrid systems. Such advances also incentivize the investigation of metal–organic frameworks (MOFs) as reparable nanocarriers with the potential to provide high loading capacity, controlled release kinetics, and easy post-synthetic modification to facilitate targeted delivery, beyond passive targeting through the enhanced permeability and retention (EPR) effect. Most advanced platforms also employ stimuli-sensitive components to initiate drug release at the tumor site with minimal leakage before reaching the site.^9,10^ Among various triggers, acidic pH is particularly advantageous for cancer-specific release. Tumors generally have an acidic extracellular pH (∼6.5–5.5) relative to normal tissues (pH 7.4), due to anaerobic glycolysis and hypoxia in the tumor microenvironment (TME).^11^ pH-Responsive nanocarriers can exploit this gradient to enable selective disintegration or pore opening, releasing their therapeutic payload precisely where it is needed.^12^

Metal–organic frameworks (MOFs) offer several advantages over conventional nanoparticles as drug carriers due to their high porosity, tunable pore size, and modular chemical composition. Their crystalline, cage-like structures enable exceptionally high drug loading capacities and controlled release profiles, while the organic–inorganic hybrid framework allows for customizable surface functionality to improve biocompatibility and targeting efficiency.^13,14^ Compared to other nanocarriers, such as liposomes, polymeric nanoparticles, or metal oxides, MOFs can encapsulate both hydrophilic and hydrophobic molecules, and their pH-responsive degradation facilitates controlled drug release in acidic tumor or endosomal environments.^15^ However, several limitations still restrict the clinical translation of MOF-based drug-delivery systems. The stability of MOFs in biological media remains a concern, as some frameworks degrade prematurely or release toxic metal ions. Therefore, while MOFs represent a highly versatile and promising new platform for precision drug delivery, their chemical stability and biological safety must be optimized for reliable therapeutic use.^16,17^

Zeolitic imidazolate frameworks (ZIFs) have received growing interest for drug-delivery applications because of their large surface area, adjustable porosity, and inherent pH sensitivity.^18,19^ ZIF-90, built out of zinc ions and imidazole-2-carboxaldehyde, is a uniquely interesting structure, especially in that its surface aldehyde groups allow easy functionalization.^20^ It is also acid-biodegradable and can be prepared as nanoparticles with sizes of less than 200 nm, a size scale that benefits tumor penetration and internalization by cells.^21^

Active targeting ligands, *e.g.* folic acid (FA), are frequently used to enhance selectivity. FA has a high affinity for folate receptors, which are overexpressed in most breast cancers, such as TNBC and HER2-positive subtypes, but are expressed at low levels in normal tissues.^22,23^ When conjugated with nanoparticles, FA brings about receptor-mediated endocytosis, enhancing the delivery of drugs to the cancer cells with minimal off-target accumulation.^24^ FA conjugation has the potential not only to stimulate receptor-mediated uptake, but can also improve colloidal stability and circulation behavior.^25^ While there is extensive literature on the use of FA-functionalized ZIF-8 platforms to deliver cargos, like doxorubicin and nucleic acids,^26,27^ there are few studies on the delivery of FA-functionalized ZIF-90, with the majority of the literature focusing on cisplatin delivery.^28^ Systematic studies of docetaxel encapsulation within FA-modified ZIF-90 are lacking.

**Scheme 1:**
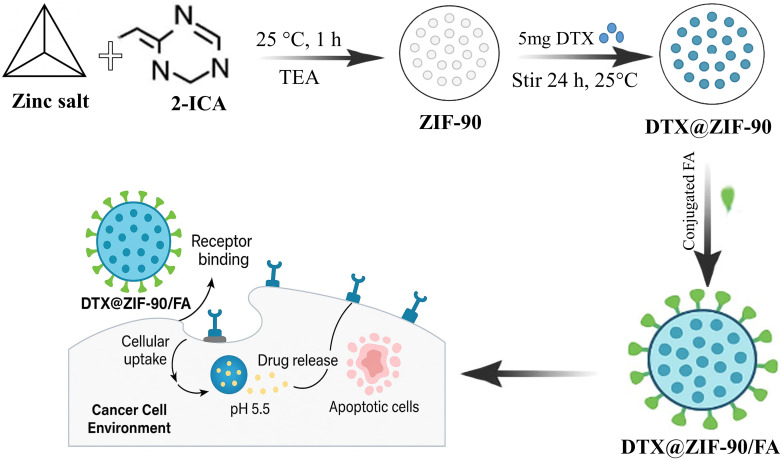
Schematic of the synthesis of DTX@ZIF-90 and induced DTX release from DTX@ZIF-90/FA *in vitro*.

To address this gap, we designed and characterized folic acid-functionalized ZIF-90 nanocarriers (DTX@ZIF-90/FA) to deliver pH-responsive docetaxel (Scheme 1). This system integrates high encapsulation performance, regulated release in the presence of acid, and FA-mediated targeting capability. To the best of our knowledge, this is also one of the earliest comprehensive reports about docetaxel encapsulation in FA-modified ZIF-90, which forms a robust material basis for future biological validation.

## 2. Experimental section

### 2.1 Materials and reagents

Zinc nitrate hexahydrate (Zn(NO_3_)_2_·6H_2_O, 99% purity), imidazole-2-carboxaldehyde (2-ICA, 97% purity), polyvinyl pyrrolidone (PVP; average molar mass = 40 000 Da), phosphate-buffered saline (PBS), absolute methanol (99% purity), triethylamine (TEA, 99% purity), hydrochloric acid (HCl), dimethyl sulfoxide (DMSO, 99% purity), and *N*,*N*-dimethylformamide, DMF (99% purity) were obtained from Sigma Aldrich. Acetone, ethanol, and folic acid were obtained from Biochem and used without further purification. Docetaxel (DTX, 98.60% purity) was obtained from Targeted Moll (Germany). All reagents and solvents were of analytical or pharmaceutical grade and used as received.

### 2.2 Synthesis of ZIF-90

To prepare ZIF-90, imidazole-2-carboxaldehyde (2-ICA, 75 mg, 2 mmol) was dissolved in 12 mL of methanol that included 0.1 mL of triethylamine (TEA). Individually, a 110 mg (1 mmol) solution of zinc nitrate hexahydrate (Zn(NO_3_)_2_·6H_2_O) was dissolved in 12 mL of methanol in a 50 mL beaker. Individual solutions were stirred for 10 min. The ligand solution was subsequently added in droplets to the solution of zinc salt under constant stirring, and heating at 75 °C was done after 30 min. This mixture was then stirred at room temperature for over 24 h, and toward the end of this time, pale-yellow precipitates appeared, indicating that ZIF-90 had successfully been produced. Upon centrifugation (10 000 rpm, 10 min), the solid product was washed carefully with methanol (5 × 3 mL) and dried at room temperature under vacuum (24 h) to obtain the as-synthesized ZIF-90 as a pale-yellow solid.^29^

### 2.3 Synthesis of DTX@ZIF-90

Docetaxel (DTX) was encapsulated into the ZIF-90 framework using a solvent-assisted impregnation technique. Firstly, 50 mg of dried ZIF-90 was suspended in methanol by sonication to obtain an even suspension. In an alternative vessel, 5 mg of DTX was dissolved in ethanol and poured dropwise into the ZIF-90 suspension with steady stirring. The resulting solution underwent sonication for 1 h, followed by magnetic stirring for 24 h at room temperature for drug loading. After loading, the nanoparticles were obtained by centrifugation, cleaned by removing the non-encapsulated drug, and dried under vacuum. Encapsulation efficacy (EE%) and drug loading capability (DLC%) had been measured spectrophotometrically at 229 nm with the aid of a standard calibration plot (*R*^2^ = 0.9978).^30^ The encapsulation efficiency (EE%) and drug loading capacity (DLC%) were quantified *via* UV-vis spectrophotometry by measuring the residual DTX concentration in the supernatant at 229 nm, using the pre-established calibration curve. Encapsulation efficiency and drug loading were calculated using the following formulas:
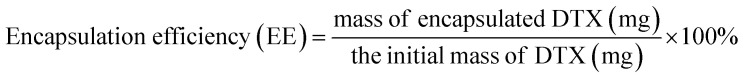

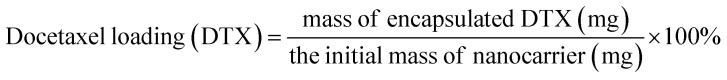


### 2.4 Post-synthesis of DTX@ZIF-90/FA

Surface functionalization of DTX@ZIF-90 with folic acid (FA) was achieved through Schiff base conjugation. DTX@ZIF-90 (50 mg) was dispersed in 10 mL of methanol under gentle stirring. Separately, folic acid (10 mg) was dissolved in 5 mL of ethanol with sonication to ensure complete dissolution. The folic acid solution was then added dropwise to the DTX@ZIF-90 suspension and stirred at room temperature for 12 h to facilitate covalent conjugation *via* imine (C=N) bond formation between the aldehyde functionalities (–CHO) of the imidazole-2-carboxaldehyde linkers and the primary amine groups (–NH_2_) of folic acid. No reducing agent was employed to preserve the pH-labile nature of the Schiff base linkage. The resultant DTX@ZIF-90/FA nanoparticles were collected by centrifugation (10 000 rpm, 10 min), washed twice with methanol to remove unbound or weakly adsorbed folic acid, and dried under ambient conditions.

### 2.5 Characterizations

Powder X-ray diffraction (PXRD) patterns were recorded using an X'Pert HighScore diffractometer, with a scanning-angle range of 5°–80° and a total scan time of 80 min. Surface morphology and particle shape were observed by scanning electron microscopy (SEM) using a TESCAN MIRA LMS system. Dynamic light scattering (DLS) measurements were performed using a Zetasizer Nano ZSE analyzer (Malvern Instruments) to determine the particle-size distribution. Nitrogen adsorption–desorption isotherms were obtained to evaluate surface characteristics, with the Brunauer–Emmett–Teller (BET) method used for surface area analysis and the Barrett–Joyner–Halenda (BJH) method for pore-size distribution. Fourier-transform infrared (FTIR) spectroscopy was conducted using a Nicolet 6700 spectrophotometer within the wavenumber range of 500–4000 cm^−1^ to identify functional groups. Zeta potential measurements were carried out by dispersing the samples in phosphate-buffered saline (PBS) and analyzing them with a Zetasizer Nano ZSE. Ultraviolet-visible (UV-vis) absorption spectra were recorded using a Shimadzu UV-2800 spectrophotometer over a 200–400 nm wavelength range.

### 2.6 *In vitro* release studies

An *in vitro* release study was carried out to analyze the pH-dependent release behavior of docetaxel (DTX) from ZIF-90-based nanocarriers under simulated physiologic and tumor-mimicking conditions. Weighed quantities of the DTX@ZIF-90 and the DTX@ZIF-90/FA nanocarriers equivalent to 1 mg of DTX were dispersed in 5 mL of the phosphate-buffered saline (PBS) solution at pH 7.4 and pH 5.5, respectively. We chose an acidic pH of 5.5 to mimic the endosomal/lysosomal compartments and the extracellular environment of the tumor during cellular internalization, and the normal blood and tissue conditions of pH 7.4 were also chosen. Each sample was moved to a pre-soaked dialysis tube (3500 Da molecular weight cut-off), sealed, and submerged in 50 mL of the PBS buffer solution containing 0.5% Tween-80, maintaining the sink conditions. The release system was kept at 37 ± 0.5 °C with gentle shaking (100 rpm) in the thermostated orbital shaker.^31^ At predetermined time intervals (0.5, 1, 2, 4, 8, 12, 24, and 48 h), 1 mL of the release medium was withdrawn and replaced with an equal volume of fresh buffer to maintain constant volume and sink conditions. The amount of released DTX was quantified using UV-vis spectrophotometry at 229 nm, and cumulative release (%) was calculated based on a standard calibration curve. Each release experiment was carried out in triplicate. The results are reported as mean ± standard deviation (SD). To better identify the mechanism of release, the measured cumulative release data were fitted with different kinetic models, such as zero-order, first-order, Higuchi, and Korsmeyer–Peppas models. The kinetic model with the highest coefficient of determination (*R*^2^) was the best fit, denoting the most appropriate mechanism of release.^32^ Nonlinear regression analysis was performed using GraphPad Prism version 9.5.1 (GraphPad Software, USA).

### 2.7 Statistical analysis

All the experiments were carried out in triplicate, except otherwise stated, and the data are expressed as mean ± SD. Statistical analysis was carried out using GraphPad Prism (software version 9.5.1, GraphPad Software Inc., USA, released in 2023). One-way analysis of variance (ANOVA) was utilized in determining differences between experimental groups, with Tukey's *post hoc* test applied for multiple comparisons. Statistically significant values were maintained at less than 0.05. All the experiments were repeated in triplicate. In release profile studies, statistical differences between formulations, as well as between pH conditions, were established to analyze the pH-response behavior of the nanocarrier system.

## 3. Results and discussion

**Fig. 1:**
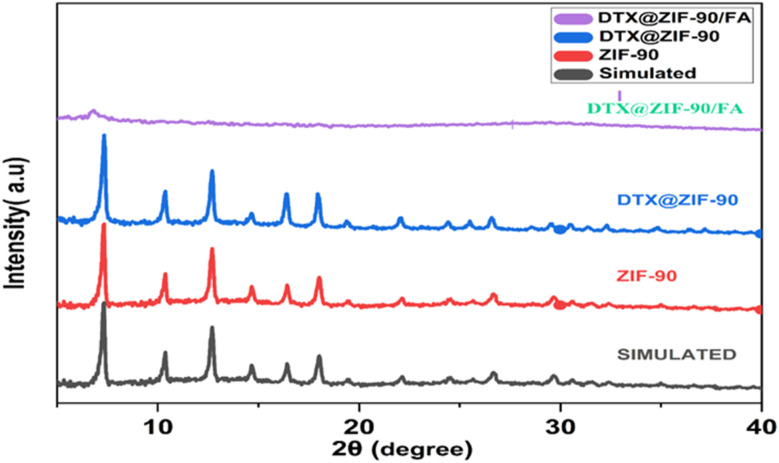
Powder X-ray diffraction (PXRD) patterns of simulated ZIF-90 (black), synthesized ZIF-90 (red), DTX@ZIF-90 (blue), and DTX@ZIF-90/FA (purple), confirming crystallinity retention and successful functionalization at each stage.

This study evaluated the synthesis, functionalization, and *in vitro* characterization of ZIF-90-derived nanocarriers for the targeted delivery of DTX. The results demonstrated the effective entrapment of the DTX within the ZIF-90 lattice, functionalization with folic acid (FA), and pH-responsive drug release. These findings are reported along with the potential for an increase in the bioavailability and therapeutic effect of the DTX. The synthesized materials were comprehensively characterized using a range of analytical techniques, including PXRD, FTIR, FESEM, BET, DLS, and zeta potential analysis, to verify their phase purity, crystalline structure, and topological and morphological features. The PXRD pattern of ZIF-90, as shown in Fig. 1, shows excellent agreement with the simulated reference pattern, confirming the successful formation of a pure ZIF-90 phase. Characteristic PXRD peaks are seen at 2*θ* values of roughly 7.4°, 10.4°, 12.8°, 14.7°, 16.4°, and 18.0°, characteristic of the sodalite-type topology, thus reaffirming the successful preparation of the highly crystalline ZIF-90 architecture.^33^ After the encapsulation with DTX, the PXRD pattern for DTX@ZIF-90 retains key diffraction features of pure ZIF-90, but it displays reduced peak intensity by a moderate degree. This attenuation suggests that DTX loading occurs predominantly within the pores of the framework, leading to partial obstruction of long-range order without significantly compromising the crystallinity of the parent structure. Following post-modification of the surface modification with folic acid (FA) to form DTX@ZIF-90/FA, the intensity for the PXRD peaks greatly diminishes with considerable peak broadening, characteristic of reduced crystallinity. This change is most likely due to the effects of the functionalization on surfaces, including the addition of an amorphous organic coat overlay, as well as potential outer-surface structural changes.^34^ Despite this reduction in peak sharpness, the residual presence of ZIF-90-specific reflections suggests that the internal framework remains partially undamaged. In summary, PXRD analysis confirms the structural integrity of ZIF-90 through successful encapsulation with DTX, resulting in FA conjugates that reduce the material's crystallinity and enhance its surface functionality for prospective trace drug delivery.

**Fig. 2:**
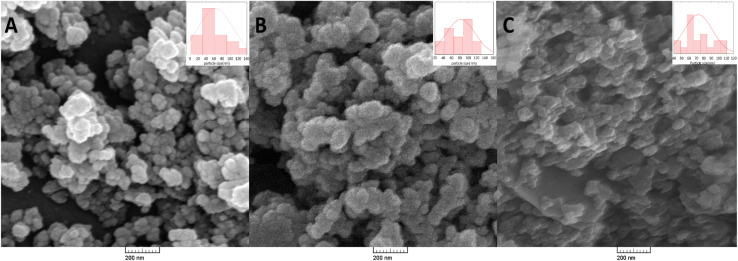
Field-emission scanning electron microscopy (FE-SEM) images and corresponding particle-size histograms (inset) of (A) pristine ZIF-90, (B) docetaxel-loaded ZIF-90 (DTX@ZIF-90), and (C) folic acid-functionalized nanocarriers (DTX@ZIF-90/FA).

Field-emission scanning electron microscopy (FESEM) images and particle-size distribution analyses provide crucial insights into the structural and morphological transformations occurring during the drug loading and surface functionalization of ZIF-90 nanoparticles (Fig. 2). From Fig. 2A, the initial ZIF-90 nanoparticles present a rather polyhedral shape with appreciable clumping. From the histogram showing the size distribution, the mean particle diameter is approximately 60 ± 12.07 nm, demonstrating a rather similar nanoscale synthesis appropriate for biomedical uses, where nanoparticles less than 100 nm in diameter are capable of permeating through the obstacles present in the body.^35^ The nanoscale size also allows for efficient targeting and drug delivery, which are crucial in cancer treatment, as nanoparticles below 100 nm can take advantage of the enhanced permeability and retention (EPR) effect in tumors.^36^ Upon drug (DTX) loading, the nanoparticle morphology changed (Fig. 2B). The particles became irregular in shape but showed minimal aggregation, a change most likely due to the sequestration of the drug within the porous ZIF-90 scaffold. This is also supported by the wider distribution, where the apex moved to 80 ± 13.52 nm, suggesting a change in the structural composition over the scaffold, possibly as the pores enlarge or the outer covering swells during drug sequestration. These findings are in line with findings by others, where the drug loading has been shown to change the particle diameter due to structural readjustments.^37^

Further surface functionalization with folic acid (FA), a known targeting ligand for folate receptor-positive malignancies, induced additional changes in morphology (Fig. 2C). The nanoparticles are less defined and rough, most likely due to the conjugation of the FA molecules to the surfaces of the nanocarriers. This further surface modification deposits an organic outer coat, enhancing the biocompatibility and targeting specificity of the nanocarriers. Notably, the distribution becomes smaller in size, with the peak now at 70 ± 30 nm, indicating that FA conjugation enhances targeting and stabilizes the nanoparticles, potentially preventing the aggregate formation observed in drug-loaded specimens.^38^ This stabilization is critical for colloidal stability under physiological conditions, as well as for maintaining the nanoparticles' activity throughout the duration of delivery. The observed changes in particle size and surface morphology at each progressive stage (pure ZIF-90, DTX@ZIF-90, and DTX@ZIF-90/FA) confirm the successful synthesis. These transformations indicate that the nanocarrier is properly equipped for enhanced drug delivery and targeting. Such results are in agreement with previous studies that have demonstrated the effectiveness of FA functionalization in enhancing the targeting efficacy of nanoparticulate drug-delivery platforms.^39^ Such transformations in particle sizes, as well as the surface properties observed through FESEM, are in agreement with the successive steps in functionalization, along with their postulated biomedical applications for drug delivery and tumor targeting.

The observed darker contrast in Fig. 2C compared to the brighter contrast in Fig. 2A can be attributed to the differences in surface composition and conductivity. Pristine ZIF-90 exhibits higher electron scattering from metallic zinc nodes, giving a brighter appearance, whereas the FA coating and organic DTX loading in Fig. 2C increase the surface carbon content and reduce secondary electron emission, resulting in a darker contrast. These morphological and contrast changes confirm the sequential drug encapsulation and FA surface functionalization, consistent with the successful synthesis of the DTX@ZIF-90/FA nanocarrier.

**Fig. 3:**
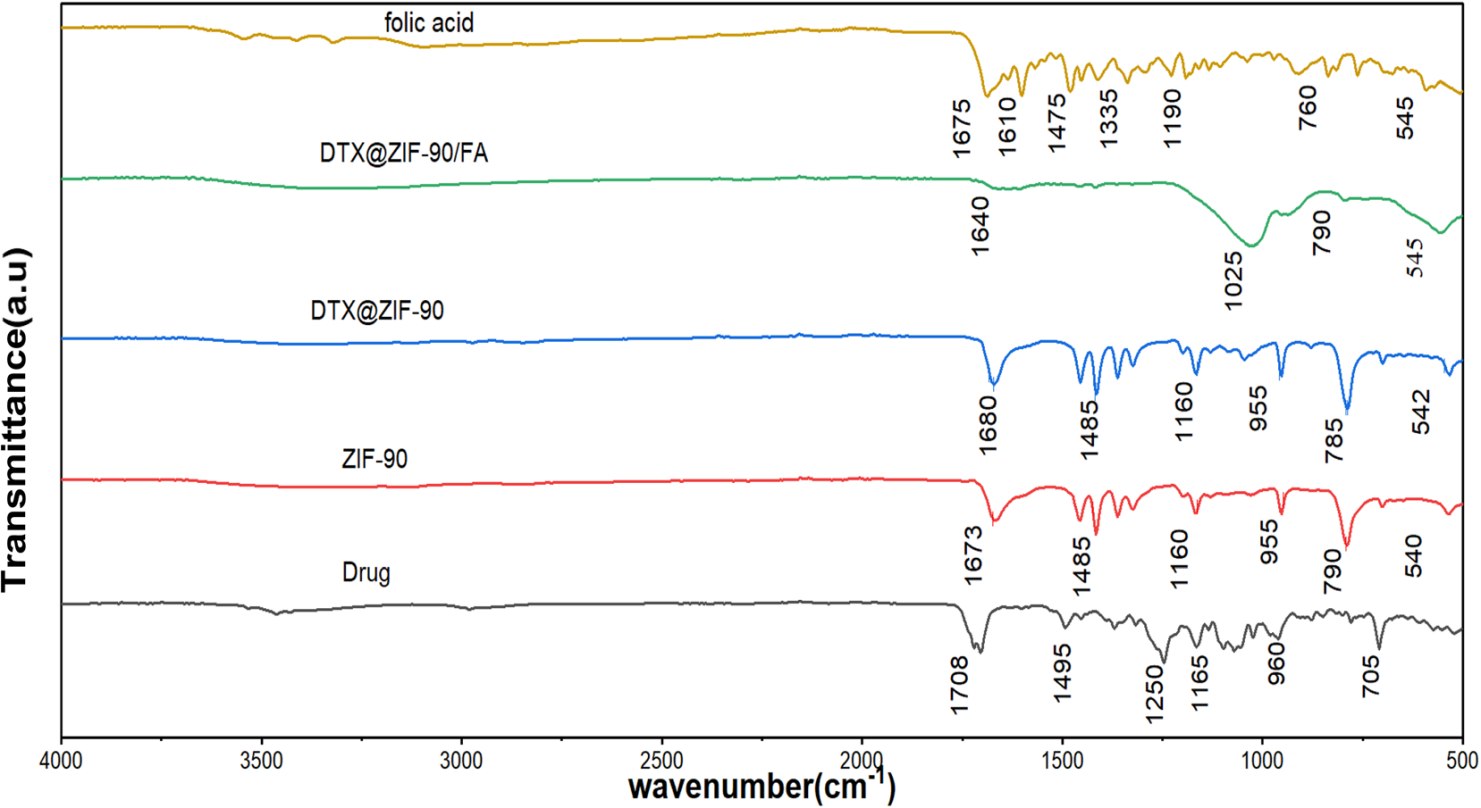
Fourier-transform infrared (FTIR) spectra of DTX (black), ZIF-90 (red), DTX@ZIF-90 (blue), DTX@ZIF-90/FA (green), and folic acid (yellow), showing the functional group interactions and confirming drug loading and FA surface conjugation.

The surface functional groups of the synthesized ZIF-90 nanocarriers were thoroughly analyzed using Fourier transform infrared (FTIR) spectroscopy, as presented in Fig. 3. The FTIR spectrum of pure ZIF-90 displays characteristic peaks at 1673 cm^−1^ (C=N), 1485 cm^−1^, and 540 cm^−1^ (Zn–N), which are indicative of the imidazole framework's integrity and confirm that the ZIF-90 structure remains intact following synthesis. These peaks correspond to the primary metal–ligand bonds within the ZIF-90 network, affirming the stability and preservation of its crystalline structure.^40^

Following the DTX encapsulation (DTX@ZIF-90), the FTIR spectrum retains the typical peaks of the parent ZIF-90. New bands are found at 1680 cm^−1^ and 955 cm^−1^, suggesting the successful incorporation of the drug inside the ZIF-90 lattice. The downshift of the C=N stretching vibration band from 1673 cm^−1^ to 1680 cm^−1^ and the appearance of the band at 955 cm^−1^ indicate the ZIF-90 framework interaction with the drug, capturing the drug inside the porous network. This evidence is in agreement with the findings of foregoing studies demonstrating similar drug–framework interactions for MOF drug delivery platforms,^41^ confirming that the DTX loading does not disrupt the overall structural integrity of ZIF-90.

Subsequent to the functionalization of the surface with folic acid (FA) (DTX@ZIF-90/FA), absorption bands at 1640 cm^−1^ (C=O) and 1025 cm^−1^ (C–O), due to the carboxyl and hydroxyl group present in FA, respectively, were observed. These bands confirm the successful conjugation of FA on the surfaces of the nanocarriers, realizing the targeting function required for selective folate receptor-positive targeting to cancer cells.^42^ The appearance of the new bands ensures the successful functionalization with FA, facilitating targeted drug delivery while maintaining the stability of the nanoparticles.

In the FTIR spectra of the pure DTX, strong bands at 1708 cm^−1^ (C=O) and 1250–1050 cm^−1^ (C–N) are observed, which correspond to the functional groups of the DTX. These bands also appear in the FTIR spectra of the DTX@ZIF-90 but with a concurrent loss, suggesting that the functional groups of the DTX are preserved after the drug has been encapsulated in the ZIF-90 structure. The preservation of these functional groups is crucial for the drug's retention of its pharmacological activity, serving as proof that the drug loading, as well as the FA surface functionalization, did not significantly alter the drug's chemical structure. In the FTIR spectrum of pure DTX, prominent bands at 1708 cm^−1^ (C=O) and 1250–1050 cm^−1^ (C–N) are observed, corresponding to the functional groups of DTX. These peaks are also present in the spectrum of DTX@ZIF-90, albeit with some attenuation, suggesting that DTX retains its functional groups after encapsulation in the ZIF-90 framework. The retention of these functional groups is critical for maintaining the pharmacological activity of DTX, confirming that both the drug loading and FA surface functionalization did not significantly alter the chemical structure of the drug.

The functionalization of DTX@ZIF-90 with folic acid was achieved through Schiff base formation, exploiting the reactive aldehyde groups (–CHO) present on the imidazole-2-carboxaldehyde linkers of the ZIF-90 framework. Multiple lines of evidence from FTIR spectroscopy, zeta potential measurements, and particle-size analysis collectively confirm the successful covalent conjugation.

The FTIR spectrum of DTX@ZIF-90/FA (Fig. 3) exhibits a characteristic shift in the C=N stretching vibration from 1673 cm^−1^ (pristine ZIF-90) to 1640 cm^−1^, consistent with imine (Schiff base) bond formation between the aldehyde groups of ZIF-90 and the primary amine groups (–NH_2_) present in the pteridine ring and glutamate tail of folic acid. This downfield shift, accompanied by band broadening, indicates successful covalent conjugation through the following reaction:ZIF-90–CHO + H_2_N–FA → ZIF-90–CH=N–FA + H_2_O

The appearance of additional bands at 1640 cm^−1^ (C=O stretching of FA carboxyl groups) and 1025 cm^−1^ (C–O stretching) further corroborates the presence of folic acid on the nanocarrier surface. The overlapping of the newly formed C=N imine stretch (typically 1620–1690 cm^−1^) with the FA carbonyl band accounts for the observed peak position at 1640 cm^−1^.

The textural characteristics of the synthesized nanocarriers were evaluated using Brunauer–Emmett–Teller (BET) analysis, based on nitrogen adsorption–desorption isotherms, to assess their suitability for drug encapsulation and release. The specific surface area, pore volume, and mean pore diameter values for the pure ZIF-90, drug-loaded ZIF-90 (DTX@ZIF-90), and folic acid-modified ZIF-90 (DTX@ZIF-90/FA) are listed in Table 1.

**Table 1:** BET surface area, pore volume, and pore diameter of ZIF-90, DTX@ZIF-90, and DTX@ZIF-90/FA^a^

Sample name	BET surface area (m^2^ g^−1^)	Pore volume (cm^3^ g^−1^)	Mean pore diameter (nm)
ZIF-90	371.54	0.578	6.23
DTX@ZIF-90	309.57	0.557	5.90
DTX@ZIF-90/FA	41.97	0.324	4.22

a Values obtained from BJH analysis correspond to inter-particle (textural) mesopores; intrinsic micropores of ZIF-90 are <1 nm.

The BET results reveal a considerable decrease in the surface area and porosity following drug loading as well as surface functionalization. The specific surface area decreased from 371.54 m^2^ g^−1^ for the pure ZIF-90 to 309.57 m^2^ g^−1^ for DTX@ZIF-90, indicating the successful encapsulation of the DTX inside the inner pores of the ZIF-90 lattice. Another substantial loss to 41.97 m^2^ g^−1^ took place for DTX@ZIF-90/FA, substantiating the additional coverage on the surface by molecules of folic acid (FA). Similarly, the pore volume decreased from 0.578 to 0.557 cm^3^ g^−1^ and then to 0.324 cm^3^ g^−1^, but the mean pore diameter fell from 6.23 nm to 5.90 nm and subsequently to 4.22 nm.

**Fig. 4:**
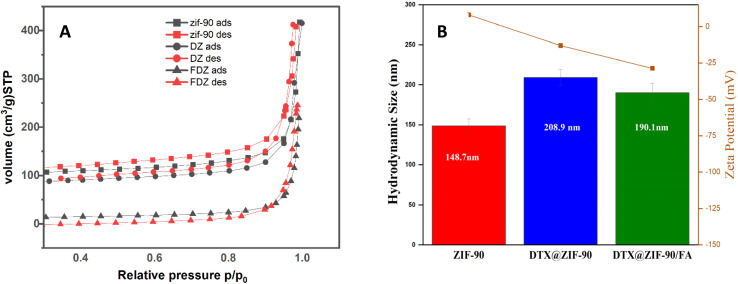
(A) N_2_ adsorption–desorption isotherms of ZIF-90, DTX@ZIF-90, and DTX@ZIF-90/FA, showing changes in the BET surface area and porosity. (B) DLS and zeta potential data indicating increased particle size after drug loading (DTX) and stabilization *via* post-folic acid (FA) functionalization.

This progressive reduction in porosity and surface area reflects the drug molecules' sequential occupation of the framework's pores and the functional blocking of surface sites by the targeting ligand (FA). These findings are in agreement with the previous reports showing that MOF-based carriers significantly reduce the surface area, as well as the accessibility of the pores, when they undergo post-synthetic functionalization and guest-molecule loading.^43^ The decrease in the textural parameters proves the successful modification of the structure. This highlights an increase in the potential for sustained drug release, as the pore constriction has the effect of retarding the drug diffusion out of the matrix. Furthermore, the nitrogen adsorption–desorption isotherm for ZIF-90 (Fig. 4A) exhibits the typical type IV isotherm with H3 hysteresis loop for mesoporous materials containing slit-like pores. This structural property aligns with the drug-delivery potential of the material due to its mesopores (2–50 nm), which enable optimal drug loading with the potential for pH-responsive release mechanisms within the tumour microenvironment.^44^

While the ZIF-90 framework is intrinsically microporous, the N_2_ adsorption–desorption profile of the synthesized nanocrystalline sample exhibited a type IV isotherm with a characteristic H3 hysteresis loop. This isotherm shape is not indicative of the framework's micropores but is a clear sign of textural mesoporosity. This mesoporosity arises from the aggregation of the nanocrystals, which creates non-uniform, slit-like interparticle voids. The pore-size distribution reported in Table 1 was calculated using the BJH method, which is specifically suited for quantifying such mesopores. Therefore, the data in Table 1 reflect this textural porosity, while the intrinsic framework micropores (<2 nm) fall below the reliable quantification range of this particular analysis method.^45–48^

Zeta potential measurements (Fig. 4B) provide complementary evidence of successful FA conjugation. The surface charge underwent a dramatic reversal from +8.0 ± 1.2 mV for pristine ZIF-90 to −28.8 ± 1.5 mV for DTX@ZIF-90/FA. This substantial negative shift is attributed to the abundant carboxyl groups (–COOH/–COO^−^) present in the glutamate moiety of folic acid. At the measurement pH, these carboxyl groups are partially deprotonated, imparting strong negative charges to the nanoparticle surface. The magnitude of the negative zeta potential (−28.8 mV) not only confirms the successful surface modification but also indicates excellent colloidal stability, as particles with |*ζ*| > 20 mV typically exhibit strong electrostatic repulsion that prevents aggregation in physiological media. This enhanced stability is crucial for maintaining particle dispersion during circulation and facilitating cellular uptake *via* folate receptor-mediated endocytosis.

Zeta potential measurements characterize dispersed nanocarriers, not free drug molecules. Pure docetaxel, being a crystalline hydrophobic solid, cannot form a stable colloidal suspension; therefore, its zeta potential cannot be directly determined. The zeta potential of pristine ZIF-90 nanoparticles was +8.0 ± 1.2 mV, which shifted to −13.1 ± 1.8 mV upon DTX encapsulation (DTX@ZIF-90), indicating surface modifications due to surface charge redistribution and the presence of adsorbed DTX molecules. Following folic acid (FA) functionalization, the zeta potential further decreased to −28.8 ± 1.5 mV, reflecting a highly negative surface charge attributed to the abundant carboxyl groups (–COOH) of FA molecules. This substantial negative charge serves multiple critical functions: (i) it enhances colloidal stability through increased electrostatic repulsion between particles, preventing aggregation in biological fluids; (ii) it facilitates electrostatic interactions with positively charged regions on cell membranes, promoting cellular uptake; and (iii) it enables specific targeting through folate receptors (FRs), which are overexpressed on many cancer cells and mediate the receptor-mediated endocytosis of FA-functionalized nanoparticles, thereby enhancing tumor-targeting efficacy.^49^

In summary, FTIR spectroscopy, zeta potential analysis, and release kinetics collectively demonstrate that folic acid was successfully conjugated to DTX@ZIF-90 *via* Schiff base formation and that the unreduced imine linkage contributes meaningfully to the system's pH-responsive behavior, positioning DTX@ZIF-90/FA as a promising dual-responsive nanocarrier for targeted cancer chemotherapy.

Dynamic light scattering (DLS) analysis (Fig. 4B) showed that the initial ZIF-90 nanoparticles exhibited an average hydrodynamic diameter of 148.7 ± 8.7 nm, indicating monodisperse particle formation. After the loading with DTX, the hydrodynamic diameter increased to 208.9 ± 10.2 nm, substantiating successful drug entrainment within the structure, resulting in an increase in the hydrodynamic size. Such an increase is typical for the nanoparticulate systems where the drug molecules are entrapped within the pores of the structure. After conjugating the nanoparticles with folic acid, the diameter decreased slightly to 190.1 ± 11.6 nm, smaller in comparison with the sample loaded with the drug. This decrease is likely due to the formation of the compact outer shell through the FA coating, which would reduce the particle aggregation and enhance the nanoparticles' stability.^50^

The polydispersity index (PDI) values of 0.160 for ZIF-90, 0.145 for DTX@ZIF-90, and 0.269 for DTX@ZIF-90/FA demonstrate the relatively narrow size distributions of the particles, indicating good colloidal stability and uniformity throughout synthesis and surface modification. Although the PDI values increase slightly following functionalization with FA, the resulting PDI value of 0.269 remains in the permissible range (PDI < 0.3); thus, the system is indeed sufficiently monodispersed for potential use in targeted drug delivery.^51^

## 4. Encapsulation, release, and kinetic study

### 4.1 Encapsulation efficiency, yield, and drug loading capacity

An initial DTX loading of 5 mg was determined to be optimal for ZIF-90 encapsulation, representing an effective compromise between maximizing encapsulation efficiency (EE%) and drug loading capacity (DLC%). This formulation achieved an EE% of 92.8%, substantially higher than values observed at elevated DTX concentrations, where framework saturation resulted in reduced encapsulation efficiency. Comparative analysis reveals that this performance exceeds those of many reported MOF carriers, particularly FA-functionalized ZIF-8 systems (typical EE%: 70–80%), underscoring the superior host–guest compatibility of ZIF-90 for hydrophobic pharmaceutical compounds. Specifically, a 5 mg dose of DTX is efficiently hosted by ZIF-90 nanoparticles, which possess intrinsic porosity and surface area. Consequently, high percentages of the drug are embedded within the carrier's matrix, as demonstrated by the high EE%. This concentration also ensures the nanoparticle carrier is not overladen with a high drug loading, an effect guaranteed to cause inefficient encapsulation with the consequence of reduced formulation stabilities. The resulting DTX@ZIF-90 nanocomposite was synthesized in a high yield of 99.35%, calculated based on the encapsulation of 4.64 mg of DTX in 50 mg of ZIF-90. This high yield results in minimal loss of material, making the nanocomposite useful for drug delivery. Few MOF-based formulations report such a high synthetic yield, which strengthens the translational potential of this platform for scalable drug-delivery applications. The DLC% for 5 mg was calculated to be 8.43%, showing the feasible drug loading of DTX in the drug-carrier system. This high EE%, along with a high drug yield, suggests the requirement for the selection of the optimal drug concentration for the nanoparticle-based drug-delivery system. Optimizations would also help elevate these findings, but the current findings establish a solid foundation for the potential use of ZIF-90 as a drug carrier for cancer management. Notably, this DLC exceeds those of many FA-functionalized MOFs reported for other chemotherapeutics, positioning DTX@ZIF-90/FA as a distinctive nanoplatform within breast cancer drug-delivery research.

### 4.2 *In vitro* release behavior

The *in vitro* release behavior of DTX from various formulations and under different pH conditions revealed significant contrasts in drug release kinetics and environmental responsiveness. A negligible DTX cumulative release (<10%) was observed for both acidic (5.5) and physiologic (7.4) pH environments over 48 h. This is as predicted by the similar release behavior exhibited in previous works.^52^ Its poor aqueous solubility and limited bioavailability in its unformulated form are well documented in pharmacokinetic literature.^53^ This comparison highlights the distinctive role of MOF-based carriers, specifically ZIF-90, in overcoming the inherent solubility and bioavailability barriers of DTX, which only a few FA-functionalized systems have directly addressed.

**Fig. 5:**
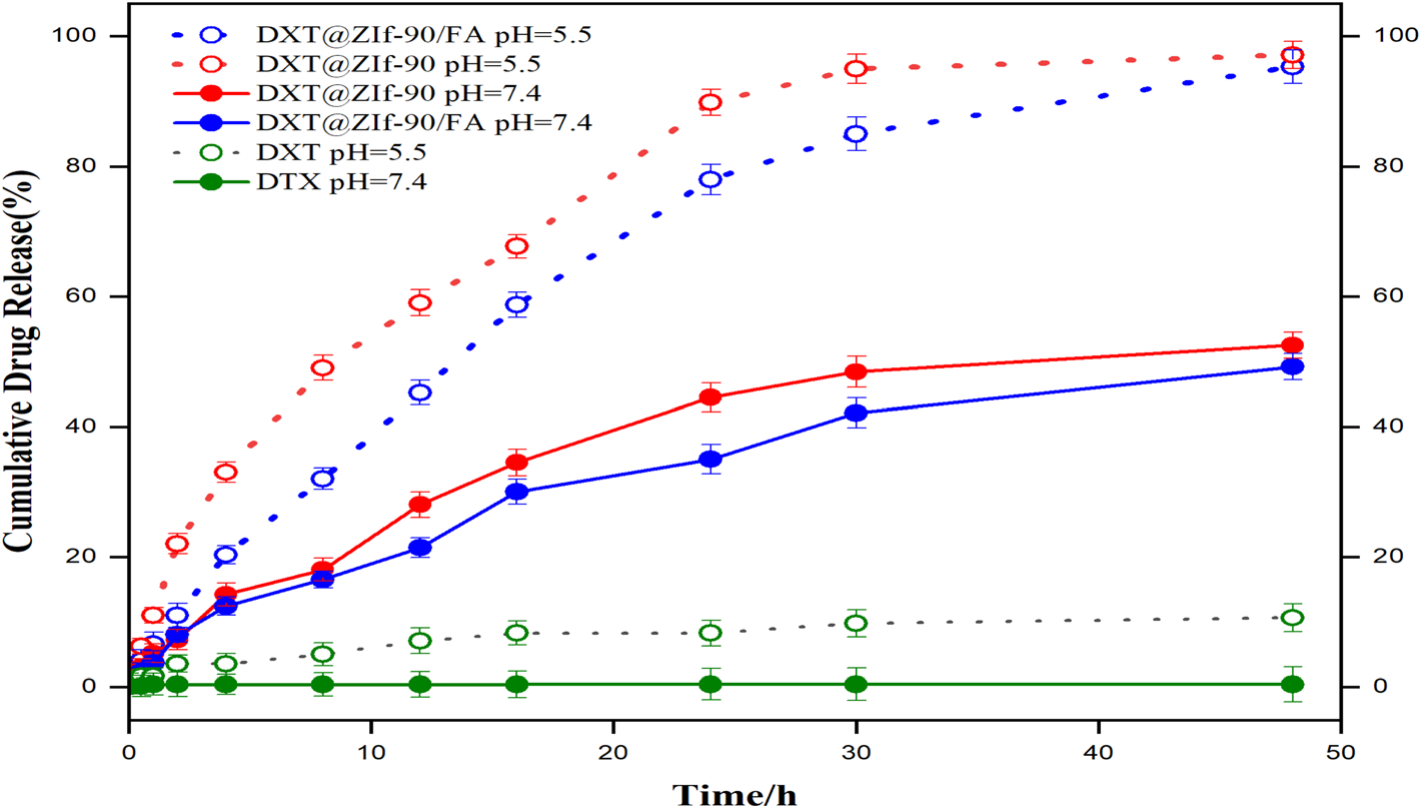
Cumulative DTX release profiles (pure DTX, DTX@ZIF-90, and DTX@ZIF-90/FA) at pH 7.4 (solid) and pH 5.5 (dashed) over 48 h at 37.5 °C. Data are presented as mean ± SD (*n* = 3). *p < 0.05 *vs.* free DTX (same pH) *via* one-way ANOVA with Tukey's test.

In contrast, DTX encapsulated within ZIF-90-based nanocarriers exhibited significantly enhanced and pH-responsive release characteristics. Specifically, the folic acid-functionalized formulation (DTX@ZIF-90/FA) exhibited an approximate 95% release at pH 5.5 over 48 h, as shown in Fig. 5, indicating successful structure disintegration and enhanced drug diffusion under tumour-mimicking conditions. This release effectiveness is comparable to those of FA-functionalized ZIF-8 formulations, where partial release and diminished pH responsiveness have been encountered, emphasizing the greater acid-responsiveness of ZIF-90 carriers. At pH 7.4, an equivalent formulation exhibited appreciably slower release (∼49%), indicating structural integrity with negligible early-time leakage under the physiological conditions. In comparison with the equivalent formulation (DTX@ZIF-90), which exhibited an equivalent accelerated release at pH 5.5 (∼98%), but appreciable, somewhat larger release at pH 7.4 (∼52%), the DTX@ZIF-90/FA formulation exhibited appreciable superiority with better responsiveness.

The pH-dependent release behavior of DTX@ZIF-90 and DTX@ZIF-90/FA arises from the intrinsic acid-labile nature of the Zn-imidazolate coordination bonds within the ZIF-90 framework. Under acidic conditions, the protonation of the imidazolate linkers weakens the Zn–N coordination interactions, leading to partial framework decomposition and the formation of enlarged diffusion pathways that facilitate accelerated drug release. In contrast, at the physiological pH (7.4), the coordination framework remains stable, resulting in slower, diffusion-controlled release. Surface functionalization with folic acid introduces additional carboxyl groups (–COOH) that undergo protonation at acidic pH, thereby increasing surface hydration and promoting matrix erosion. These combined mechanisms account for the significantly faster release observed at pH 5.5 compared to that at pH 7.4.^54^

The improved selectivity of FA-functionalized ZIF-90 is particularly novel, as most of the preexisting FA-MOF works focus on increased uptake rather than showing proof of improved release selectivity between tumor-like and physiological conditions. In both cases, the release exhibited a biphasic nature, characterized by an initial quick release or burst release during the initial 8 h, which was notable due to the drug adsorbed onto the surfaces, followed by an equivalent sustained release as the drug diffused through the porous MOF network. One-way ANOVA with Tukey's post-test (*p* < 0.05) confirmed that the drug release from the equivalent nanocarriers was significantly better than that of free DTX in an acidic medium, validating their functional responsiveness and appreciable potential for targeted tumour therapy in acidic microenvironments. Statistically confirmed biphasic, pH-responsive release is rarely reported for FA-MOF systems, making the present study an original contribution. Generally, the pH-responsive kinetics, along with the dual-phase behaviour of ZIF-90/FA, enable its promising potential for controlled site-specific DTX delivery. The appreciable pH-responsive and biphasic release behaviours of the equivalent ZIF-90 with its folic acid-derivatized counterpart (DTX@ZIF-90/FA) highlight its potential for use as an advanced nanocarrier for effective drug delivery. Specifically, the inclusion of folic acid has a significant impact on the formulation, affording an exciting targeting strategy for site-specific tumour chemotherapies in the acidic microenvironment typical of bulk solid tumours.^55^ We know of no prior systematic report of a folic acid-modified ZIF-90 docetaxel carrier that offers both high encapsulation efficiency and mechanistic release modelling, which differentiate it from prior MOF-based drug delivery platforms.

### 4.3 Kinetic modeling of drug release

The *in vitro* release kinetics of DTX from various formulations were systematically investigated under physiological pH (7.4) and acidic pH (5.5) conditions using classical mathematical models to elucidate the underlying release mechanisms. As shown in Table 2, five kinetic models (zero-order, first-order, Higuchi, Hixson–Crowell, and Korsmeyer–Peppas) were applied to the cumulative release profiles of free DTX, DTX@ZIF-90, and folic acid-functionalized DTX@ZIF-90/FA nanocarriers.^56^ In the drug formulations, an optimum fit with the Hixson–Crowell model (*R*^2^ = 0.9969, *K* = 1.5829) was obtained for the DTX@ZIF-90/FA formulation at pH 5.5, suggesting that matrix erosion and geometric disintegration control the drug release in acidic, tumour-mimicking environments.^57^ Similarly, the optimum fit for the DTX@ZIF-90 formulation at pH 5.5 was the first-order (*R*^2^ = 0.9881, *K* = 0.0838), suggesting concentration-controlled release, with structural instability also playing a role in the low-pH environments.^58^ At physiological pH (7.4), both ZIF-90 formulations fit best with the Korsmeyer–Peppas model, with DTX@ZIF-90/FA yielding an *R*^2^ = 0.9868 and *n* = 0.5851 and DTX@ZIF-90 yielding an *R*^2^ = 0.9670 with *n* = 0.5505. Such *n* values imply anomalous (non-Fickian) transport, so that the combined effect of an interplay between diffusion and nanocarrier relaxation or erosion would control the drug release. Higuchi's model also highly correlated these systems (*R*^2^ > 0.96), substantiating the relevance of diffusion-controlled mechanisms at neutral pH. In comparison, free DTX at pH 5.5 exhibited weak fitting for all models, with the highest *R*^2^ values being less than 0.97 and the *n* value being 0.4033 (Korsmeyer–Peppas). This suggests traditional Fickian diffusion with no structural modulation, attributed to the poor aqueous solubility of the DTX and the absence of a carrier system to act as an intermediary for controlled release.^59^

**Table 2:** Kinetic parameters of DTX released from DTX@ZIF-90 and DTX@ZIF-90/FA or as a free drug using different models

Model	Zero-order kinetics		First-order kinetics		Higuchi		Korsmeyer–Peppas			Hixson–Crowell	
Formulation/medium (pH)	*K*	*R* ^2^	*K*	*R* ^2^	*K*	*R* ^2^	*K*	*R* ^2^	*n*	*K*	*R* ^2^
DTX@ZIF-90/FA pH 5.5	2.5428	0.8411	0.0564	0.9942	14.1268	0.9617	10.2865	0.9722	0.5992	1.5829	0.9969
DTX@ZIF-90 pH 5.5	2.8224	0.638	0.0838	0.9881	16.2136	0.9648	18.0255	0.9597	0.4665	2.2725	0.9808
DTX@ZIF-90 pH 7.4	1.442	0.7807	0.0218	0.9234	8.1016	0.9669	6.8977	0.967	0.5505	0.6401	0.8885
DTX@ZIF-90/FA pH 7.4	1.2667	0.8434	0.0177	0.9399	7.0449	0.9777	5.3682	0.9868	0.5851	0.5308	0.9155
Free DTX pH 5.5	0.3008	0.4307	0.0032	0.4778	1.7486	0.9325	2.3703	0.9655	0.4033	0.1053	0.4624
Free DTX pH 7.4	0.0149	−5.0845	0.0001	−5.076	0.0969	−1.896	0.3531	0.3737	0.0747	0.005	−5.0791

The pH-dependent release mechanisms are validated by kinetic modeling analysis. The release profiles at pH 5.5 best fit the Hixson–Crowell model (*R*^2^ = 0.9969), confirming the erosion-dominated release resulting from acid-induced framework decomposition. In contrast, the release at pH 7.4 follows the Korsmeyer–Peppas model (*R*^2^ = 0.9155), indicating diffusion-controlled release through the intact framework. These distinct kinetic behaviors validate the dual-mechanism pH-responsive release: rapid erosion-mediated release under acidic conditions mimicking the tumor microenvironment and slow diffusion-controlled release at physiological pH. Collectively, these findings demonstrate that the folic acid-functionalized ZIF-90 nanocarriers exhibit pH-responsive, controllable drug release governed by both environmental pH and carrier architecture, rendering them highly promising for targeted and sustained chemotherapeutic delivery in cancer therapy.^60^

## 5. Conclusion

This work describes the synthesis and overall characterization of pH-responsive, folic acid-functionalized ZIF-90 nanocarriers loaded with docetaxel (DTX@ZIF-90/FA). PXRD, FTIR, FESEM, and BET structural studies were used to verify the maintenance of the integrity of the framework after surface modification and drug loading. The encapsulation efficiency of the nanocarrier was very high (92.8%), but the drug loading capacity was also significant (8.43%). *In vitro* release studies showed a strong sensitivity to pH, with increased release in acidic conditions mimicking tumour microenvironment compared with that at the physiological pH.

Kinetic modelling also helped put into context specific release mechanisms: the Hixson–Crowell model (*R*^2^ = 0.9969) modeled erosion-dominated release in the pH = 5.5 regime, and the Korsmeyer–Peppas model (*R*^2^ = 0.9155) modeled diffusion-controlled release in the pH = 7.4 regime. These mechanistic observations offer uncommon and precious data on surface erosion and unusual transport phenomena in FA-modified MOF drug systems. As far as we understand, this work is among the earliest to systematically demonstrate the encapsulation of docetaxel in FA-functionalized ZIF-90 nanocarriers, unlike the better-investigated ZIF-8 analogues.

Future work will focus on the biological efficacy of DTX@ZIF-90/FA using folate-receptor-mediated uptake, cytotoxicity, and apoptosis assays with breast cancer models, as well as *in vivo* biodistribution to evaluate its suitability as a targeted therapeutic platform.

## Author contributions

Rizgar Noori: conceptualization, methodology, investigation, writing – original draft. Nian N. N. Maarof: supervision, project administration, writing – review & editing. Azad H. Alshatteri: supervision, validation, formal analysis, writing – review & editing.

## Conflicts of interest

The authors declare no competing interests.

## Data Availability

All data supporting the findings of this study are included within the article. Additional raw data (including PXRD, FTIR, BET, FESEM, DLS, and drug release profiles) are available from the corresponding author upon reasonable request.
